# Supplementary material for: Anti-HLA Class II Antibodies Correlate with C-Reactive Protein Levels in Patients with Rheumatoid Arthritis Associated with Interstitial Lung Disease
Source: Cells. 2020 Mar 11;9(3):691. doi: 10.3390/cells9030691 (PMC7140697; doi:10.3390/cells9030691)
Supplement: Supplementary file 1 [file cells-09-00691-s001.pdf]

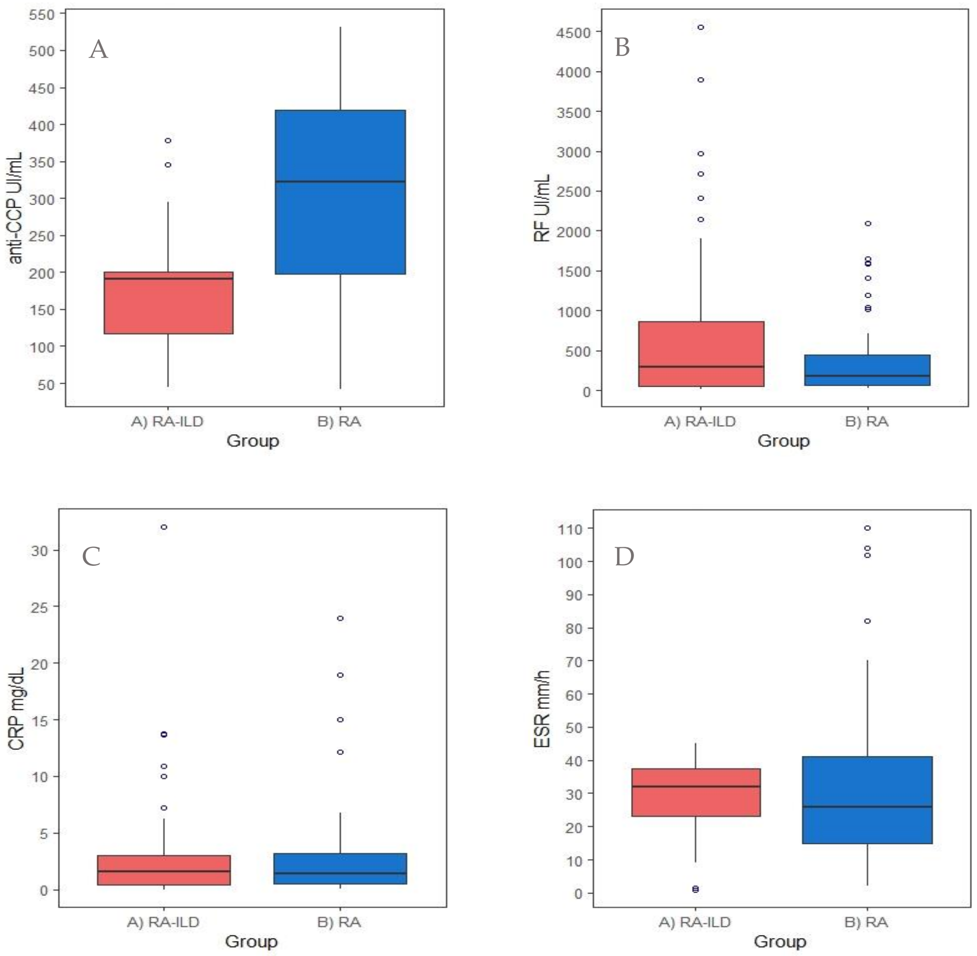

Supplementary Figure 1. The levels of biomarkers of the RA with or without ILD in 147 patients. The comparison between groups no statistical differences were found. (A) Levels of anti-CCP (UI/mL), (B) Levels of FR (UI/mL), (C) Levels of CRP (mg/dL) and (D) Levels of ESR (mm/h).

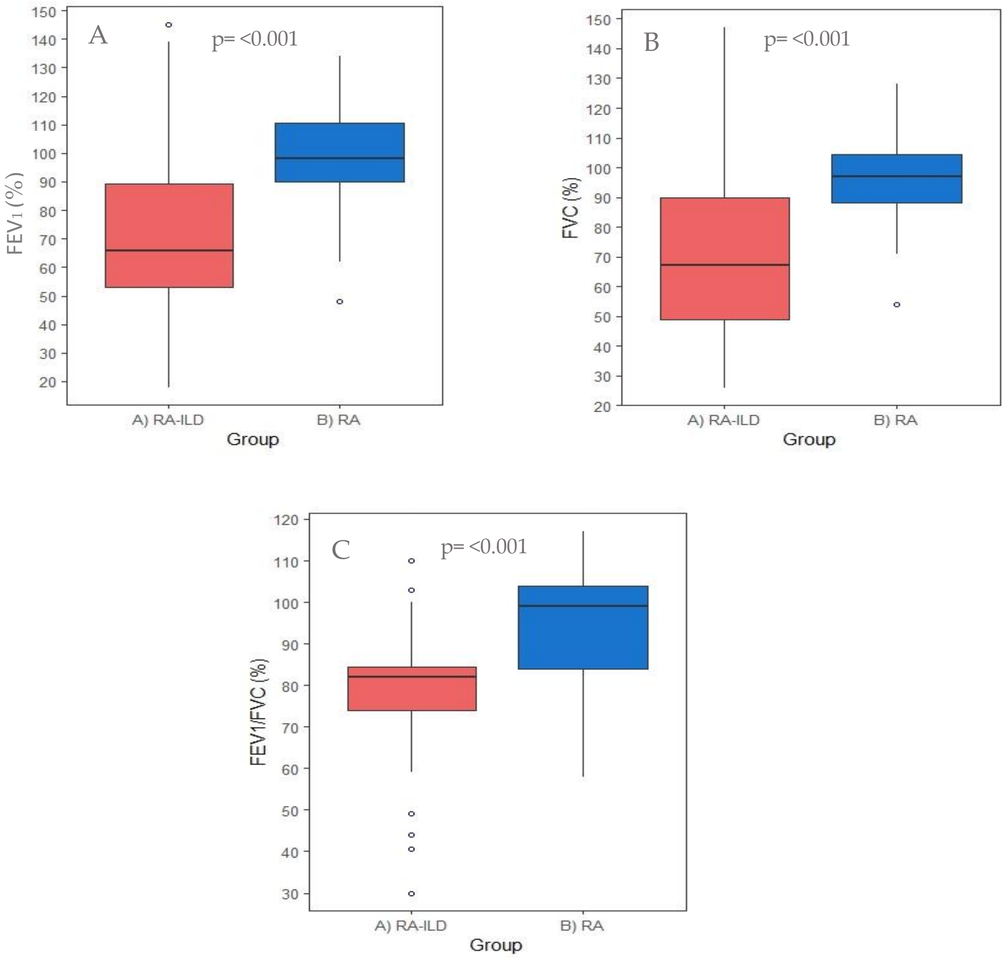

Supplementary Figure 2. The percentage of lung function values of the RA with and without ILD in 147 patients. The comparison between groups statistical differences were found. (A) FEV_1 (%): forced expiratory volume in the first second values, (B) FVC (%): forced vital capacity values and (C) FEV_1/FVC (%): forced expiratory volume in the first second/forced vital capacity ratio.

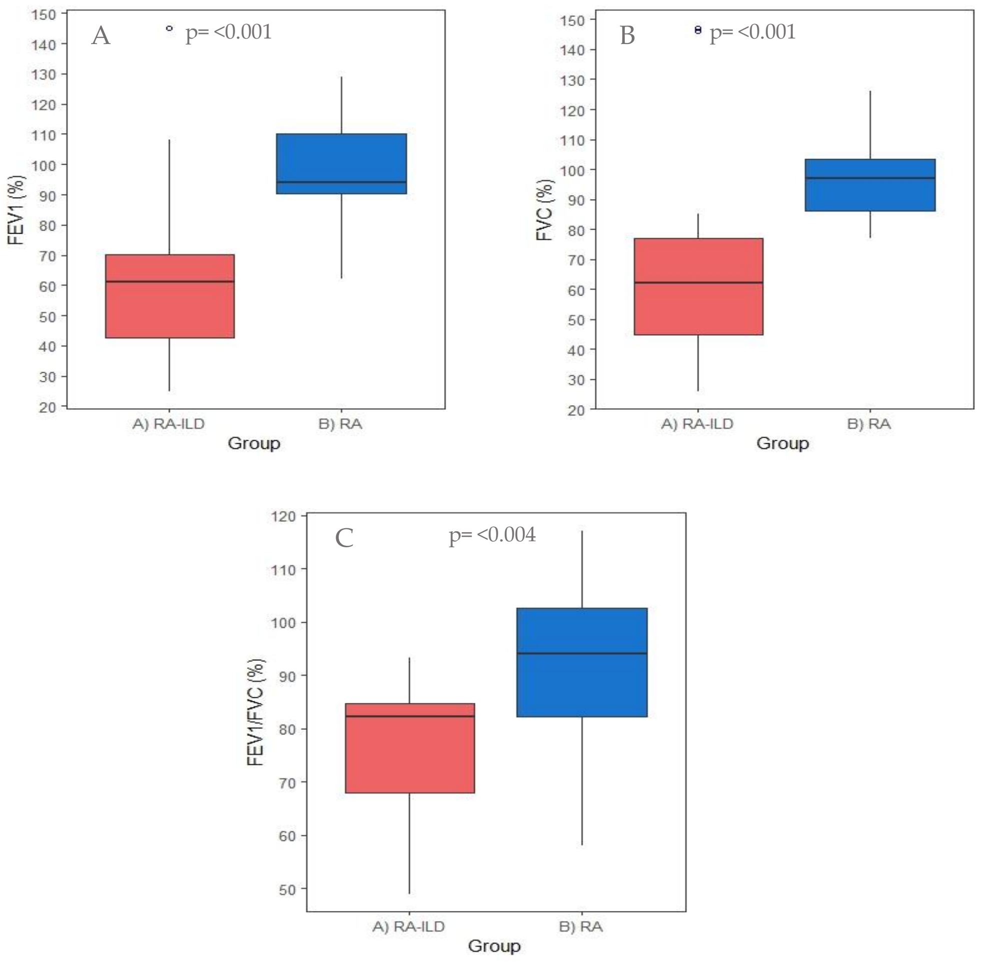

Supplementary Figure 3. The percentage of lung function values of the RA with and without ILD in 147 patients. The comparison between groups statistical differences were found. (A) FEV_1 (%): forced expiratory volume in the first second values, (B) FVC (%): forced vital capacity values and (C) FEV_1/FVC (%) forced expiratory volume in the first second/forced vital capacity ratio.

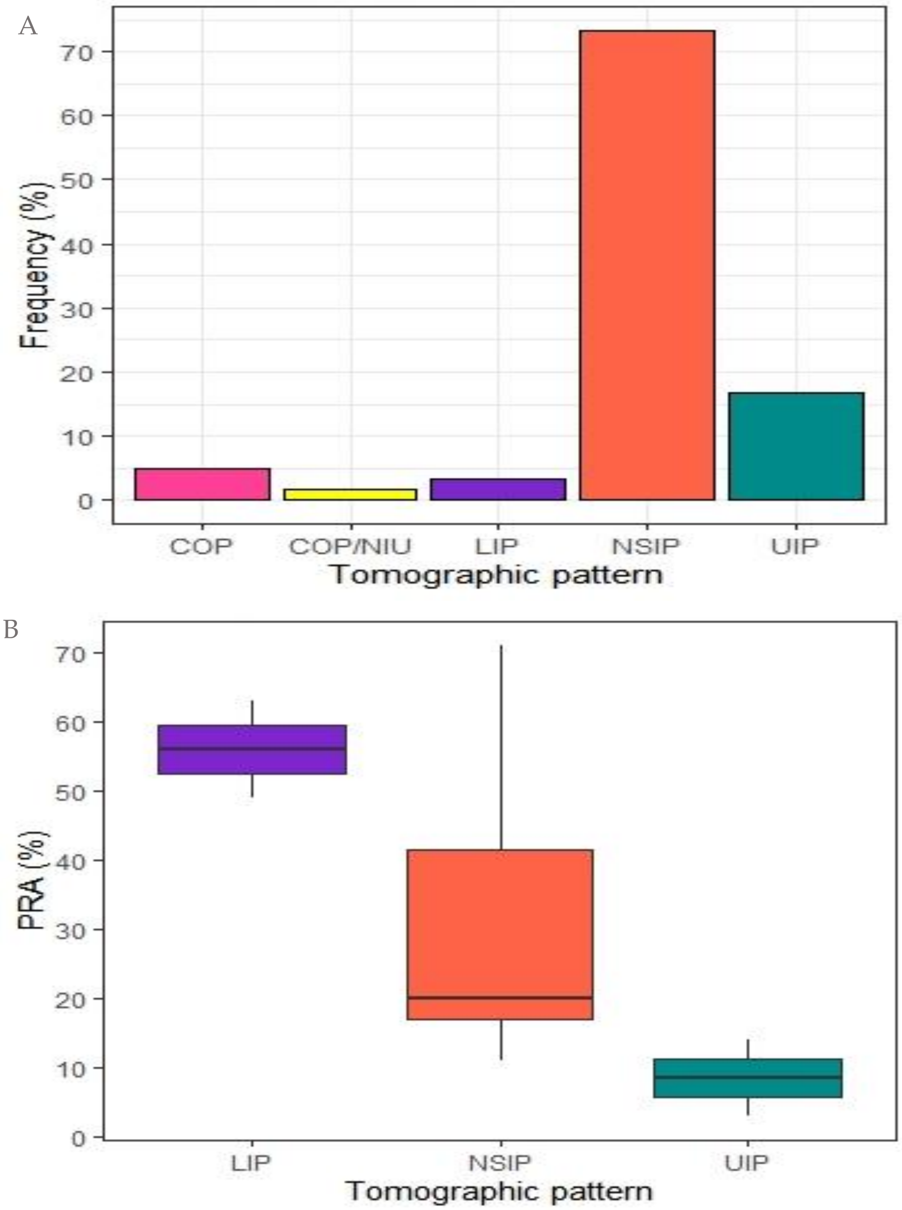

Supplementary Figure 4. Tomographic pattern in patients with ILD-RA. A) Tomographic patterns found in patients with ILD. B) Tomographic patterns found in patients with ILD positive for PRA

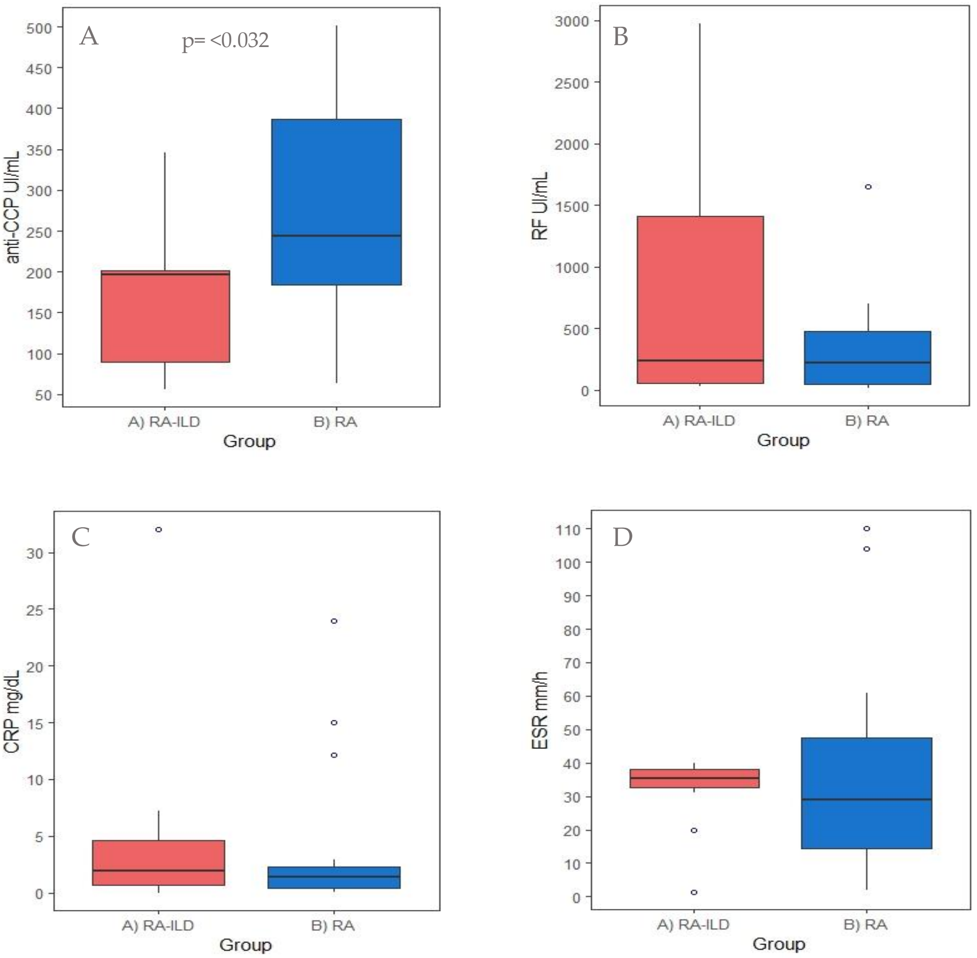

Supplementary Figure 5. The levels of biomarkers of the RA with or without ILD in patients PRA positive. The comparison between groups no statistical differences were found. (A) Levels of anti-CCP (UI/mL), (B) Levels of FR (UI/mL), (C) Levels of CRP (mg/dL) and (D) Levels of ESR (mm/h).
